# Deconstruction of the (Paleo)Polyploid Grapevine Genome Based on the Analysis of Transposition Events Involving *NBS* Resistance Genes

**DOI:** 10.1371/journal.pone.0029762

**Published:** 2012-01-11

**Authors:** Giulia Malacarne, Michele Perazzolli, Alessandro Cestaro, Lieven Sterck, Paolo Fontana, Yves Van de Peer, Roberto Viola, Riccardo Velasco, Francesco Salamini

**Affiliations:** 1 IASMA Research and Innovation Centre, Fondazione Edmund Mach, S. Michele all'Adige, Trento, Italy; 2 Department of Plant Systems Biology, VIB, Gent, Belgium; 3 Department of Plant Biotechnology and Bioinformatics, Ghent University, Gent, Belgium; The Centre for Research and Technology, Hellas, Greece

## Abstract

Plants have followed a reticulate type of evolution and taxa have frequently merged via allopolyploidization. A polyploid structure of sequenced genomes has often been proposed, but the chromosomes belonging to putative component genomes are difficult to identify. The 19 grapevine chromosomes are evolutionary stable structures: their homologous triplets have strongly conserved gene order, interrupted by rare translocations. The aim of this study is to examine how the grapevine nucleotide-binding site (NBS)-encoding resistance (*NBS-R*) genes have evolved in the genomic context and to understand mechanisms for the genome evolution. We show that, in grapevine, i) helitrons have significantly contributed to transposition of *NBS*-*R* genes, and ii) *NBS*-*R* gene cluster similarity indicates the existence of two groups of chromosomes (named as Va and Vc) that may have evolved independently. Chromosome triplets consist of two Va and one Vc chromosomes, as expected from the tetraploid and diploid conditions of the two component genomes. The hexaploid state could have been derived from either allopolyploidy or the separation of the Va and Vc component genomes in the same nucleus before fusion, as known for Rosaceae species. Time estimation indicates that grapevine component genomes may have fused about 60 mya, having had at least 40–60 mya to evolve independently. Chromosome number variation in the Vitaceae and related families, and the gap between the time of eudicot radiation and the age of Vitaceae fossils, are accounted for by our hypothesis.

## Introduction

Plants have followed a reticulate type of evolution: in their natural history, taxa have frequently merged because of polyploidization events [1]–[3]. Although component genomes are known in some polyploid crops [4], in other taxa even the cytological approach may not resolve genome components. Because genome sequences are available [1], [5], [6], transposition events which have created large gene families [7], such as the nucleotide-binding site (NBS)-encoding resistance (*NBS*-*R*) genes, could be analyzed. If component genomes have been kept separated before a polyploidization event during evolution, the transposition event may be restricted to a fraction of the extant genome, and this would allows us to recognize the old and recent history of the species.

*NBS*-*R* genes encode proteins with a nucleotide-binding site as part of the so-called NB-ARC domain [8] and sometimes with a leucine-rich repeat domain (LRR) [9], [10]. NBS*-R* proteins may have, as an amino terminal sequence, a toll/interleukin-1 receptor (TIR) domain or a coiled-coil (CC) structure [11]. The NB-ARC domain is proposed to function as molecular switch that controls the activation state of the protein, and the other domains play role in defining pathogen recognition specificity and downstream signalling [8]. *NBS*-*R* genes occupy single loci or are organized in clusters [12]. In the latter case, gene duplication via unequal crossing over has been demonstrated to have the capacity to generate the clusters [13], [14]. *NBS*-*R* gene clusters may include paralogous sequences giving rise to heterogeneous clusters [15], [16]. Duplication of chromosomal segments hosting *NBS*-*R* genes or clusters has also been reported [17].

Thus, an extensive analysis of *NBS*-*R* gene organization can increase the understanding of the evolution of a complex polyploid genome. The problem in such an approach is that, although genome duplication leading to polyploidy has played a major role in angiosperm evolution [2], [18], ancestral linkage groups tend to be dispersed on many rearranged chromosomes, with genomes having suffered wholesale gene losses [19], [20]. Such evolutionary changes in structure and number of chromosomes make it difficult not only to find a direct link between whole genome duplication (WGD) and ploidy state of a species [21], but also to recognize the founders of polyploid genomes.

Grapevine chromosomes, however, appear stable from an evolutionary point of view. Grapevine chromosomes can be easily assorted in triplets because an unexpected within triplet gene order has persisted for many tens of millions of years [6], [22]. Because of this, transposition events can be analysed in grapevine in the absence of confounding effects caused by chromosomal translocations and fragment duplications [23].

In this paper, cluster similarity, phylogenetics, and transposition events of *NBS*-*R* genes have been studied to evaluate alternative hypotheses of how the triplicate state of the grapevine genome has evolved.

## Results

### *NBS*-*R* genes and clusters: chromosome grouping

The grapevine Pinot Noir genome contain 391 predicted *NBS*-*R* genes, of which 346 have been anchored to the genome. Of the anchored *NBS*-*R* genes, 55 are single and 291 are grouped into 52 clusters (CL), each consisting of 2 to 15 genes separated by an average distance of 8.3 kilo bases (kb) (Table S1 and Table S2). Clusters extend from 3.6 to 742 kb and, on average, include 7 non-*NBS* open reading frames. *NBS*-*R* genes preferentially map on chromosomes 1, 3, 5, 7, 9, 12, 13, 15, 18 and 19. *CC*-type *NBS*-*R* genes predominate: 111 have the *LRR* domain (*CC-NBS-LRR*) and 32 lack the *LRR* domain (*CC-NBS*). Among all *NBS*-*R* genes, 27 have the *TIR* and *LRR* domains (*TIR-NBS-LRR*), 6 have the *TIR* domain (*TIR-NBS*), 145 have the *LRR* domain (*NBS-LRR*) and 70 have only the NB-ARC domain (*NBS-tr*). Of the 29 anchored *TIR*-type genes, 23 are clustered and are exclusively located on chromosomes 1, 5, 12, 13 and 18 (Table S3).

Comparisons among 346 anchored *NBS*-*R* genes generated 23693 Ks values, indicating synonymous substitutions per synonymous site. Of those, 22779 values are between genes of different clusters and still not in cluster (single *NBS*-*R* genes), denoted as Ks between genes (Ks-bg). Ks-bg was therefore used to estimate the rate of synonymous substitutions between transposed *NBS*-*R* genes that could give rise to two different clusters during evolution. The remaining 914 Ks values were derived from comparisons between genes of the same cluster (denoted as within clusters, Ks-w) and indicated the rate of synonymous substitutions between genes of the same cluster. Ks-bg scores had a mean of 1.75, while Ks-w scores had a mean of 0.90 (Figure S1). A comparison of means and distributions of Ks-bg and Ks-w support the inference that genes of the same cluster originated mainly by tandem duplication [17].

**Figure 1:**
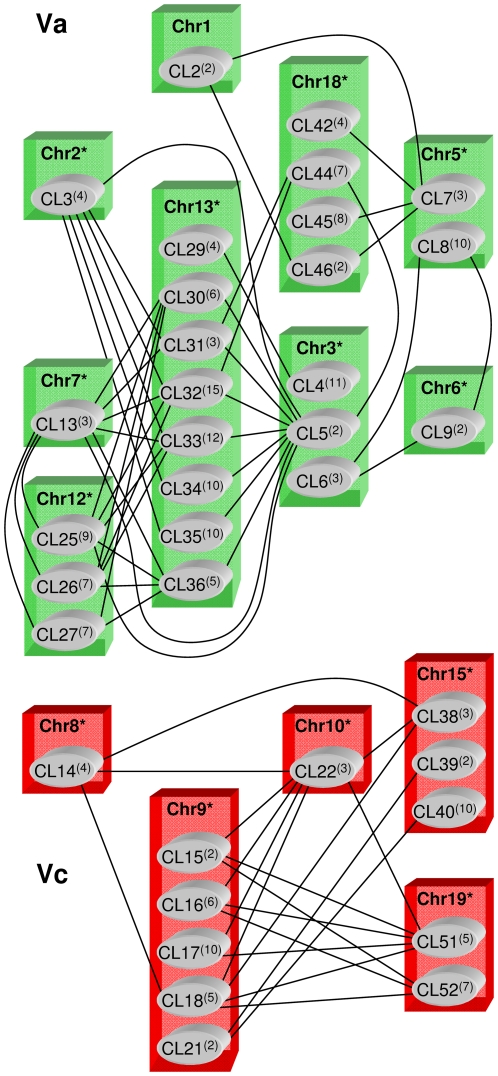
Grapevine chromosome groups based on *NBS*-*R* gene-to-gene similarities. Relationships (indicated by joining lines) between grapevine chromosomes based on the 93rd percentile of the distribution of among-clusters BLAST bit scores generated by a pairwise BLAST-P protein sequence comparison. Va (green) and Vc (red) define two genomes based on the chromosome groups indicated. Chromosomes that were also associated with Va or Vc genomes based on the identity scores are highlighted by an asterisk. *NBS*-*R* gene clusters (CL) are indicated within gray circles with the number of *NBS*-*R* genes in brackets.

Gene-to-gene similarities were also calculated as BLAST bit scores and a similarity score between clusters was developed (details in Table S4). The 93rd percentile threshold of all the between-cluster scores revealed the existence of 94 comparisons (out of 1326) and made it possible to visualise *NBS*-*R*-based similarities among grapevine chromosomes (Figure 1). High cluster similarities denoted two chromosome groups: the first, indicated with Va, included chromosomes 1, 2, 3, 5, 6, 7, 12, 13, and 18, while the second, indicated with Vc, included chromosomes 8, 9, 10, 15, and 19. Because of poor content of clustered genes (Table S2), it was not possible to assign chromosomes 4, 11, 14, 16, or 17 to either group. When the more restrictive 96th percentile was used, chromosome 1 was excluded from Va group, while Vc did not change (Figure S2A). When the 90th percentile was used, few similarity bridges were detected between the two chromosomes groups, and chromosome 11 indicated a tendency to associate with Vc chromosomes (Figure S2B).

Va and Vc grouping was supported by the identity scores derived from a global alignment between the NBS-R proteins using the Needleman and Wunsch algorithm (chromosomes with asterisks in Figure 1). Based on 14 of 19 grapevine chromosomes, our results supported the hypothesis that *NBS*-*R* gene cluster formation may have followed separate routes in at least two different genomes, one putatively tetraploid (Va) and the second diploid (Vc).

### *NBS*-*R* gene phylogeny and the Va and Vc component genomes

If the Va and Vc component genomes evolved separately for a sufficient period of time, *NBS*-*R* clusters in a phylogenetic tree should tend to occupy topologies specific for each of the two putative genomes. Conversely, in presence of high gene transposition rates manifested by the extant number of *NBS*-*R* genes, a random distribution of *NBS*-*R* genes is expected if all extant grapevine chromosomes have always been included in the same nucleus.

**Figure 2:**
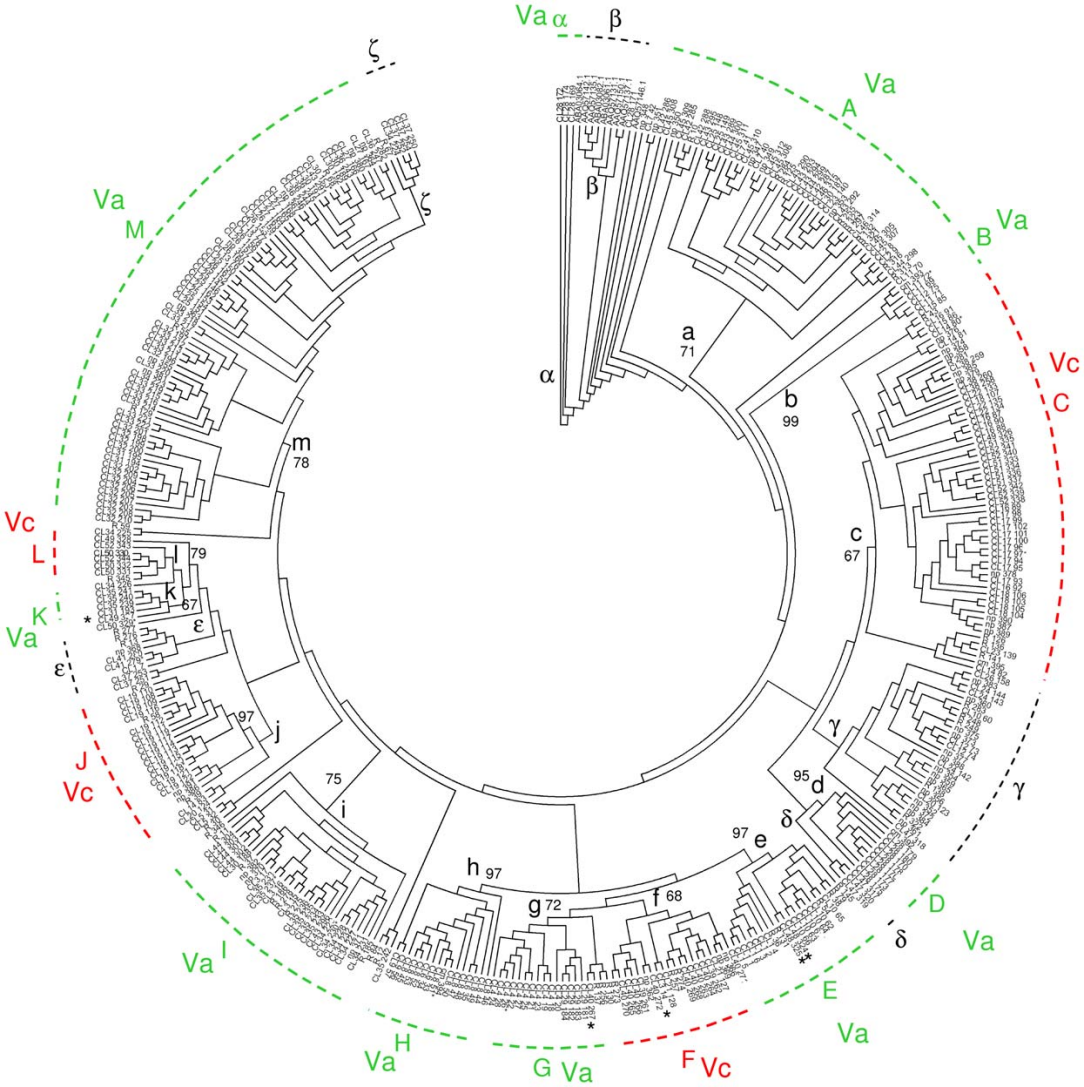
Phylogenetic analysis of grapevine *NBS*-*R* genes. The most abundant *NBS*-*R* gene classes were distributed among the different clades as follows: clades B, C, D, I and J included 80, 84, 88, 55, and 73% of *CC-*type genes, respectively; clade A included 70% of *TIR-*type genes; clades E, K, and M included 55, 60, and 59% of *NBS-LRR* genes, respectively; clades F, G, and H included 90, 63, and 67% of *NBS-tr* genes, respectively. Clades were assigned to Va (green) and Vc (red) genomes based only on clustered genes (Table 1 and Table S5). Asterisks mark clustered genes considered as exceptions to the genome assignment of a specific clade. Bootstrap values for clades A to M are expressed as percentages. The analysis included seven outgroup *NBS*-*R* genes of *Pinus monticola* [24].

In a NJ phylogenetic tree based on the NB-ARC protein domain, 13 major clades (A to M) were found, and these were specific for either Va or Vc genomes (Figure 2, Table 1 and Table S4). Six additional subclades (α to ζ) were observed as singularities, with few cases of disagreement with the rule specified above. They corresponded to: subclade α (three Va genes of cluster CL28 located at the root of the tree); subclade β (one additional gene of cluster CL28 and seven outgroup *NBS*-*R* genes of *Pinus*); subclade γ (genes that were not clustered or not chromosome assigned together with nine Va- and Vc-clustered genes); and subclade ε (six Va- or Vc-clustered genes, three non-clustered genes, and one unassigned gene). Subclades δ and ζ (both with three genes) should be considered exceptions to the Va-Vc specificity rule.

**Table 1:** Presence of clustered and single (in brackets) *NBS*-*R* genes in putative Va and Vc component genomes.

Genome	Clade	*NBS*-*R* gene classes						Genes in the alternative genome
		*CC-NBS*	*CC-NBS-LRR*	*TIR-NBS*	*TIR-NBS-LRR*	*NBS-LRR*	*NBS-tr*	
Va	A	-	-	17 (3)	3	9 (1)	1	(2) *TIR-NBS-LRR*, (2) *TIR-NBS*, (1) *NBS-tr*
	B	3	1	-	-	1	-	-
	D	7	-	-	-	1	-	-
	E	2 (1)	-	-	-	7 (2)	3	(2) *NBS-LRR*, 2 *NBS-tr*
	G	1	1	-	-	4	9	(1) *NBS-LRR*, 1 (1) *NBS-tr*
	H	-	-	-	-	5	10	-
	I	11 (1)	2 (1)	-	-	8 (2)	-	(1) *NBS-LRR*
	K	2	-	-	-	2	-	1 *NBS-LRR*
	M	16	3	-	-	38 (3)	7 (1)	1 *NBS-LRR*
Vc	C	23 (1)	13	-	-	4	3	(1) *CC-NBS-LRR*
	F	-	-	-	-	1	12 (1)	(1) *NBS-tr*
	J	9	2	-	-	3	3	(2) *CC-NBS-LRR*, (1) *CC-NBS*
	L	1	-	-	-	3	2 (1)	-

Genes of clades A to M of Figure 2, as divided in classes based on presence/absence of their specific domains, are considered.

The topology of gymnosperm outgroup *NBS*-*R* genes points to Va clades α and A as the oldest from an evolutionary perspective [24]. Moreover, clades A to M include genes located in more than one chromosome, but these chromosomes always belong to either group Va or Vc (Table S5). In all chromosomes associated with Vc, at least one cluster of the genes mapping to clade C is present (Figure 2 and Table S6). In general, genes of the same cluster have almost contiguous tree topologies, as expected if local gene tandem duplication was the mechanism generating clusters [19], [25]–[27].

The plotting of chromosomes and gene clades against gene classes provided further circumstantial evidence of the existence of Va and Vc genomes: two *NBS*-*R* gene classes were Va-genome specific, and these were *TIR-NBS-LRR* and *TIR-NBS* genes (four Vc single *TIR*-type genes are discussed later). Also, clade M, which consists of *NBS-LRR* genes, tends to be associated with Va genome (Table 1, Table S4 and Table S5). The subclade distribution of the few *NBS*-*R* genes belonging to chromosomes that are not assigned to any component genome (genome-unassigned chromosomes) is reported in Table S7.

### Genome duplications

**Figure 3:**
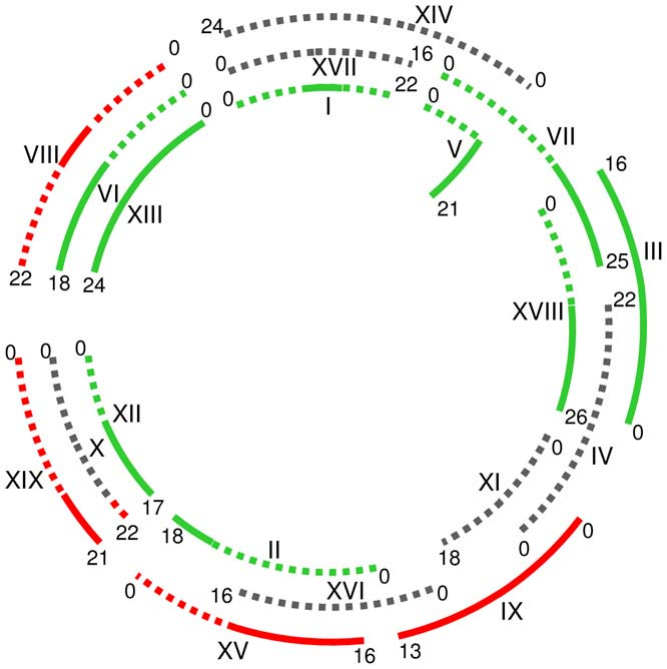
Triplicate state of *Vitis* chromosomes. The formation of triplets is based on the homology among groups of genes located on specific chromosome fragments and homologous DNA segments present on other chromosomes [5], [6], [22]. Assignment of chromosomes to either Va (green) or Vc (red) genomes was based on cluster i) similarity and ii) identity scores. Grey lines represent chromosomes not assigned to the two genomes; they could represent a third genome (Vb), for which we lack direct proof. Roman numbers indicate chromosomes, while Arabic numbers represent the length of the chromosomes in megabase pairs. Exceptions to the assignment of chromosomes are represented by chromosome IV and XIV, which could not be definitively assigned to one of the putative genomes.

Based on within-genome collinearity Jaillon et al. [6] previously showed that the grapevine genome has a triplicate structure. We have used the same approach to define grapevine chromosome triplets and have assigned chromosomes to either the Va, green (g), or the Vc, red (r), genomes (Figure 3 and Figure S3). If the ancestral Va and Vc genomes can indeed be distinguished from one another, each chromosome triplet should consist of two Va and one Vc chromosomes (assigning a tetraploid condition to the larger Va genome). In Figure S3, grey (y) indicates genome-unassigned chromosomes. Of 10 possible combinations of triplets with different colours (g, r, y), only five have been found (Figure S3) and these are triplets of: “2g and 1r”, “1g, 1r and 1y”, “1r and 2y”, “1g and 2y”, and “2g and 1y”. All these combinations, together with the combination “3y”, are compatible with the hypothesis that each triplet should consist of one Vc and two Va chromosomes. No triplet matched the hypothesis of incompatible combinations of chromosomes “1g and 2r”, “1y and 2r”, “3r”, and “3g”, with the exception of the triplet of chromosomes 10, 12, and 19 and a portion of green triplets of chromosomes 3, 7, and 18 (Figure S3). However, the assignment of chromosome 10 to the Vc genome was based on the *NBS*-*R* genes of cluster CL22, which maps at the very end of the chromosome, a position which may have been recently acquired because of chromosome end transpositions, as described for rye [28]. Based on dot plot analysis (as reported for apple by Velasco et al. [29]), the region of chromosome 10 hosting cluster CL22 is not orthologous to either chromosomes 12 or 19. For this reason, only the tip of chromosome 10 is coloured red in Figure 3.

### Expansion of *NBS*-*R* genes and clusters

**Figure 4:**
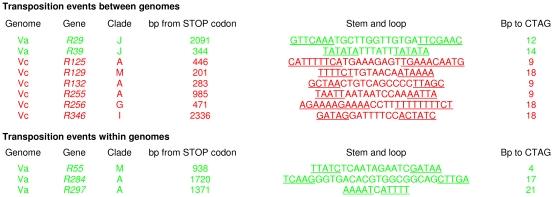
*NBS*-*R* genes showing features of residual helitron activity. The CTAG tetranucleotide and of the inverted repeat forming a stem and loop structure (underlined) are shown. The numbers of nucleotides between the two gene regions are also indicated.

Gene expansion mediated by transposition is revealed by considering single *NBS*-*R* genes. Genes *R125*, *R132*, *R255*, and *R321* (clade A) map to Vc chromosomes 9, 10, 15, and 19, respectively (Table 2 and Table S1). Because *NBS*-*R* clusters of clade A are absent in Vc, these genes could represent transpositions from Va clusters to Vc chromosomes. The complete sequence of the four Vc genes was compared to that of all Va genes: gene *314* (CL46, chromosome 18) had the lowest Ks, and we therefore assigned to it the highest probability to be the progenitor of the four putatively transposed gene copies (Table 2). The five genes mentioned above have contiguous phylogenetic topologies (Figure 2). In addition and as expected for genes transposed by helitrons [30], [31], their DNA sequence reveals, at the expected position, the CTAG motif and the inverted repeats that form a stem and loop structure (Figure 4). Also, the genes *R10*, *R284*, and *R297*, which map to Va genome, belong to clade A and have a low Ks score with the gene *314.* In *R284* and *R297*, the helitron footprints are present: they also should derive from intra-Va genome transpositions (Figure 4).

**Table 2:** Estimated time of transposition of *NBS*-*R* genes.

Single *NBS*-*R* genes	Ancestor *NBS*-*R* gene	Helitron footprints	Ks	Mya
*R125* (Vc)	CL46_*314* (Va)	+	1.34	67
*R132* (Vc)	CL46_*314* (Va)	+	1.23	62
*R255* (Vc)	CL46_*314* (Va)	+	0.69	35
*R129* (Vc)	CL32_*208* (Va)	+	0.94	47
*R346* (Vc)	CL35_*237* (Va)	+	1.89	95
*R256* (Vc)	CL4_*22* (Va)	+	1.23	62
*R39* (Va)	CL19_*113* (Vc)	+	0.77	39
*R29* (Va)	CL19_*113* (Vc)	+	0.52	26

Ks values of two genes were used to infer time following Schranz and Mitchell-Olds [3].

Va and Vc are putative component genomes of grapevine.

Similar analyses were conducted for *R79* and *R131* of clade E, *R256* of clade G, *R346* of clade I, and *R129* of clade M. All map to Vc chromosomes, and clustered genes of the corresponding clades are present only in Va chromosomes (Table 1). Of these single genes, *R256, R346,* and *R129* have putative helitron fingerprints (Figure 4 and Table S8), and their ancestors could be, respectively, clustered genes *22* (CL4) for clade G, *237* (CL35) for clade I, and *208* (CL32) for clade M. Also the gene *R55* of clade M, specific to Va genome, has the helitron footprints and could derive from the putative ancestor *208* (CL32) by an intra-Va genome transposition.

Similar results were obtained for Va genes *R29*, *R39*, and *R58* (clade J). Clustered genes of this clade were present only in Vc genome (Table 1), and among these gene *113* of cluster CL19 was found the most similar to the three single genes. Single genes *R29* and *R39* also have helitron footprints (Figure 4).

Table S8 summarizes the role of helitron-mediated gene transposition in the origin of single *NBS*-*R* genes. Of the single genes listed in the table (excluding those marked with n.d.) 29.4% should have apparently resulted from helitron-mediated transposition.

The Va to Vc transposed *NBS*-*R* genes can be used to estimate the time from their transposition, i.e., the date when their component genomes fused. Ks values from progenitor genes and their helitron-mobilized copies were converted to time values using the algorithm described by Schranz and Mitchell-Olds [3], and the estimated time did not exceed 67 mya, with one exception (*R346*, 95 mya, Table 2). The same algorithm was used to predict the time necessary for a transposed *NBS*-*R* gene to generate the homogeneous clusters present in the grapevine genome (Table 3). The two most different genes in a cluster were compared, and the resulting Ks values transformed into mya values. The calculated values ranged from 1 to 138 mya, values which indicate the estimated time for cluster formation starting from the ancestor genome to the present time.

**Table 3:** Estimated time of homogeneous *NBS*-*R* cluster formation.

Cluster	Gene number	Gene_1	Gene_2	Ks	Mya
CL2 (Va)	2	CL2_*8*	CL2_*9*	0.68	34
CL7 (Va)	3	CL7_*41*	CL7_*42*	2.32	117
CL8 (Va)	10	CL8_*51*	CL8_*48*	1.31	66
CL27 (Va)	7	CL27_*162*	CL27_*168*	1.58	79
CL29 (Va)	4	CL29_*181*	CL29_*183*	0.54	27
CL32 (Va)	15	CL32_*197*	CL32_*209*	2.74	138
CL33 (Va)	12	CL33_*220*	CL33_*222*	2.59	130
CL36 (Va)	5	CL36_*245*	CL36_*247*	0.40	20
CL42 (Va)	4	CL42_*286*	CL42_*287*	1.92	97
CL45 (Va)	8	CL45_*308*	CL45_*311*	2.55	128
CL46 (Va)	2	CL46_*314*	CL46_*315*	1.63	82
CL4 (Va)	13	CL4_*21*	CL4_*21*	2.32	116
CL6 (Va)	3	CL6_*33*	CL6_*34*	0.92	46
CL9 (Va)	2	CL9_*56*	CL9_*57*	0.38	19
CL11 (Va)	4	CL11_*69*	CL11_*70*	0.98	49
CL13 (Va)	3	CL13_*76*	CL13_*78*	0.91	46
CL15 (Vc)	2	CL15_*85*	CL15_*86*	0.085	4
CL16 (Vc)	6	CL16_*88*	CL16_*91*	2.52	127
CL17 (Vc)	10	CL17_*98*	CL17_*97*	1.11	56
CL18 (Vc)	5	CL18_*105*	CL18_*107*	2.01	101
CL19 (Vc)	15	CL19_*116*	CL19_*122*	1.21	61
CL21 (Vc)	2	CL21_*127*	CL21_*128*	1.35	68
CL22 (Vc)	3	CL22_*135*	CL22_*133*	1.08	54
CL38 (Vc)	3	CL38_*258*	CL38_*260*	0.57	29
CL39 (Vc)	2	CL39_*261*	CL39_*262*	0.01	1
CL48 (Vc)	2	CL48_*324*	CL48_*325*	0.08	4
CL51 (Vc)	5	CL51_*334*	CL51_*335*	0.65	33
CL24 (na)	3	CL24_*143*	CL24_*144*	0.95	48
CL41 (na)	2	CL41_*279*	CL41_*280*	0.78	39

Ks values of two genes were used to infer time following Schranz and Mitchell-Olds [3].

Va and Vc are putative component genomes of grapevine.

na: indicates clusters belonging to chromosomes not assigned to Va or Vc genomes.

## Discussion

### Duplication and transposition of *NBS*-*R* genes

A prominent role of tandem duplication of *NBS*-*R* genes, which was previously demonstrated for several plants [17] including grapevine [19], is supported by the low Ks values of comparisons within clusters in the current study. The formation of a gene cluster at a specific locus should be preceded by gene transposition, and selection for disease resistance may have been involved in cluster evolution [32]–[34]. A question remains concerning the formation of heterogeneous *NBS*-*R* gene clusters. It is difficult to explain the finding of *NBS*-*R* clusters that contain genes with different function-specific domains. This finding, however, may also be explained by transposition: we report a direct role of helitrons in grapevine gene mobilisation, but in plants the same role has been reported for other transposons [35], [36]. Although helitrons have the capacity to capture different transcribed genes in a single chimaeric DNA [30], [31], it remains unknown how they can assemble domains of different *NBS*-*R* genes and also relocating the new genes into existing clusters of the same gene family.

### Model for the evolution of the *Vitis* genome

Fossil seeds of Vitaceae are common in Tertiary floras [37]. Their absence in the Cretaceous suggests that the family failed to leave a fossil record or that it had not yet evolved. Fossil records strongly support the inference that the family radiated quickly at about the time of the Paleocene-Eocene transition, around 55 mya [38]. Another factor that should be considered is that Vitaceae seed remains in rocks are very reliable fossil indicators, such that their presence has a low probability to pass unrecorded [37]. Taken together, these support the inference that Vitaceae emerged around 60 mya [39]. However, molecular phylogenetic analysis indicates that the position of Vitaceae is basal to the eurosids [21], [23], [40], [41]. It is well known that modern angiosperms, after appearing in the early Cretaceous (late Barremian-early Aptian, [40], [42], see also Text S1A), rapidly diversified: within the first 10–20 million years of the early Cretaceous all major lines of flowering plants were present [18], [42]–[46]. If monocots and eudicots diverged around 150 mya [40], [43], [44], and if rosids and asterids diverged shortly thereafter (Text S1B), we would conclude that the hexapolyploidy state by the *Vitis* ancestors occurred close to 100 mya [47], [48]. At this time, eudicot angiosperms were established in geographically widespread regions as evident from tricolpate pollen grains in sediments [49]. This is why the data of Chen and Manchester [37] pose a dilemma: did the Vitaceae family emerge 60 or 100 mya?

**Figure 5:**
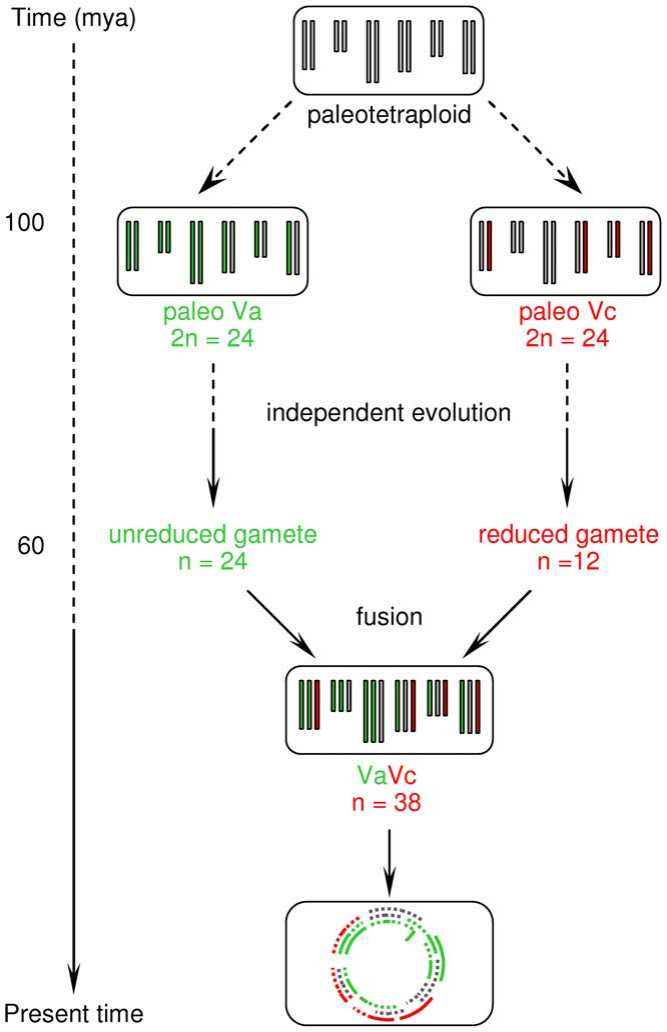
Hypothesized time of events for the evolution of the *Vitis* genome. The evolution of this genome is proposed to have followed two steps leading to hexaploidisation starting from a paleotetraploid state. In the text, a second scenario is discussed in light of the cytological data for the *Rosa canina*. The same two steps can be hypothesised, with Va and Vc genomes first coexisting in the same nucleus but not exchanging genetic material, and later fusing to generate the hexaploid state. In such a case, polyploidization probably occurred at an intermediate time between 100 and 60 mya.

A possible explanation of this dilemma can be obtained by reconsidering how the grapevine genome acquired the polyploid state. Up to 50–80% of angiosperms have a recognised hybrid origin [50], [51] and all extant angiosperm species are ancient polyploids [21], [52]. Jaillon et al. [6] discovered that three genomes contributed to the *Vitis* lineage and concluded that the polyploidy of the genome was derived from paleohexaploid ancestors. However, the alternative explanation could be the hypothesis that eudicot ancestors had a different ploidy state as recently proposed by Abrouk et al. [40]. Synthetic events leading to hexaploidy may, in fact, correspond in time to the Vitaceae emergence based on fossils. A similar hypothesis has been proposed to explain conflicts between plant molecular ages and the fossil records for crown-group *Hedyosmum* (Chloranthaceae) and for *Ephedra* (Gnetales) (Text S1A). The taxon *Hedyosmum* experienced two phases of diversification: an early Cretaceous radiation followed by a mid-Cenozoic one that generated the extant diversity [53]. Following a similar model for Vitaceae, an early evolution may have later been integrated by crosses with a species that evolved separately for a significant amount of time (Figure 5). A fusion between genomes with different ploidy has also been proposed for rosids based on a SynMap approach [54], although pre-rosid paleopolyploid events were not dated in that study. During the second phase the family may have acquired the seed morphological innovation that persists today. In support of this hypothesis we report multiple circumstantial proofs: i) the *NBS*-*R* gene cluster distribution; ii) the Va- or Vc-specific nature of most major phylogenetic clades; iii) the genome specificity of clade C (Vc) and of *TIR-NBS-LRR* and *TIR-NBS* genes (Va); and iv) the time of transposition events among Va *NBS*-*R* gene clusters and Vc chromosomes and vice versa. Our alternative hypothesis does, apparently, fit the distribution of chromosome number in extant Vitaceae genera. If the ploidy number of Vitaceae is a multiple of 6 or 7 [55], genera like *Tetrastigma* (n = 11, 22), *Cyphostemma* (n = 11) and *Cissus* (n = 12, 24, 40) have tetraploid taxa; others, *Vitis* included, have n = 19, 20 and can be considered hexaploid (even octoploid when n = 30 to 40; Text S1C). Moreover, families that are very closely related to Vitaceae, like Leeaceae, Celastraceae, Dilleniaceae and Rhamnaceae, all have almost tetraploid genera (n = 10 to 13). In conclusion, the cytogenetics of this group of related genera and families does not negate the hypothesis that their ancestors may have been tetraploid. In polyploids, moreover, genomes can minimize cytological exchanges based on mechanisms similar to the one of the *Rosa canina* complex. These pentaploid Rosaceae species have one diploid highly homozygous bivalent-forming genome and several haploid, univalent-forming homologous genomes [56], [57]. Because the intergenomic exchange of DNA is extremely poor [56], genomes separately present in the same nucleus retain their integrity. This may have represented a second possible way in which Va and Vc chromosome groups remained separated in the same grapevine nucleus before combining to form the current hexaploid genome.

That *NBS*-*R* gene clusters are Va- or Vc-chromosome specific cannot be attributed to a defective intergenomic transposition: relatively recent transpositions of single *NBS*-*R* genes between Va and Vc chromosomes are documented here. Indeed, transposition is the obvious rule in *NBS*-*R* gene and cluster evolution [7]. The rule assumes a random distribution of genes and clusters on all chromosomes. The finding of a nonrandom distribution supports the conclusion that the concerned chromosomes were initially separated and later fused in the same nucleus. Based on rough calculations, the fusion occurred around 65 mya, while the formation of *NBS*-*R* gene clusters may have started 138 mya. Both estimates agree with what is currently accepted for angiosperm evolution [40]. That chromosome pairing in *Vitis* is restricted to bivalents [55] does not contradict our conclusion: recent allopolyploid somatic hybrids [58] may have only bivalents, and in the hexaploid *Triticum aestivum* the gene region *Ph1* (*Pairing homoeologous*) suppresses multivalent formation and leads to disomic inheritance [59], [60].

### Concluding remarks

This paper, which identifies putative component genomes of *Vitis vinifera*, shows that gene transposition has the potential to dissect a complex polyploid genome. In plants, *NBS*-*R* gene duplication, as supported by gene transposition, has been a frequent event. After transposition at a new genetic locus, *NBS*-*R* gene clusters have probably been generated by tandem gene duplication. Based on *NBS*-*R* cluster similarity, we inferred the existence of two chromosome groups (named as Va and Vc) as component genomes of the extant grapevine genome. Each putative component genome is characterized by unique phylogenetic *NBS*-*R* clades and specific events of transposition, mediated particularly by helitrons, supporting the conclusion that they have evolved independently. Time estimation indicates that component genomes may have fused about 60 mya, having had at least 40–60 mya to evolve independently. The known assembly of the grapevine chromosomes in triplets enabled us to assign a tetraploid and a diploid condition to Va and Vc component genomes, respectively. The current state of grapevine hexaploidy could derive from an allopolyploidy event that occurred after eudicot radiation, or from the fusion of two genomes that were kept separated in the same nucleus during evolution.

## Materials and Methods

### Similarity analyses of genes and clusters

The grapevine Pinot Noir genome release 3 contain 391 predicted *NBS*-*R* genes (http://genomics.research.iasma.it/), [5]. The *NBS*-*R* sequences were identified based on their NB-ARC domain profile (PF00931) [61] using Hmmer [62] and were classified according to InterPro database (http://www.ebi.ac.uk/interpro/).

BLASTP on the NBS-R protein dataset retained paralogous gene pairs that could be aligned over at least 150 amino acids (identity score >30%, [63]). Based on a CLUSTALW nucleotide alignment of *NBS*-*R* gene sequences, a total of 23693 Ks values were obtained [64], with Ks values decreasing as gene similarity increased. Those values denoted as Ks-bg were derived from the pairwise comparisons between *NBS*-*R* genes of different clusters and of single *NBS*-*R* genes, and they were used to estimate the evolutionary difference between putatively transposed genes. Ks values denoted as Ks-w were derived from comparisons between genes of the same cluster.

The *NBS*-*R* gene cluster definition followed Arabidopsis rules [16]: two or more *NBS*-*R* genes were assigned to a cluster when located within an average of 244 kb, and when not interrupted by more than 21 open reading frames encoding non-NBS proteins. This cluster definition agrees well with Yang et al. [19] which used 200 kb as a distance between two contiguous *NBS*-*R* genes.

### Phylogenetic and sequence analyses

The maximum-likelihood phylogenetic tree (based on 500 bootstrap values) was constructed with PHYML [65], considering only the NB-ARC aminoacid sequence (295 aa) and using the JTT-F matrix of ML distances as the starting topology. Domains were included in clusters of protein sequences using the CD-HIT program [66], and a representative sequence was identified for each cluster. Core multiple sequence alignments (MSAs) were obtained using MAFFT [67] and extended by adding the sequences of other clusters based on T-COFFEE [68]. Seven *Pinus monticola NBS*-*R* genes [24] were included as outgroups.

### Va and Vc component genomes

A pairwise BLAST-P analysis of the complete protein sequence of 346 chromosome-anchored *NBS*-*R* genes generated gene-to-gene similarities as BLAST bit scores. Because of the time required for duplication events, clustered *NBS*-*R* genes could be used to evaluate ancient evolution events. Between-cluster BLAST bit scores were calculated on the average of n×k gene BLAST bit score comparisons, where n and k represent the number of genes of two different clusters. To select clusters significantly more similar among them, thresholds corresponding to the 90th, 93rd and 96th percentile of all scores were considered. The 93rd threshold corresponded to a mean score of 1330 BLAST bit units and selected, from a total of 1326 cluster comparisons, 94 cases of clusters having genes that were molecularly very related. The E-value of the 93rd percentile was lower than E^−300^, equal to the probability that similarity scores were due to a random association of grapevine genes.

Using the Needleman and Wunsch algorithm with the BLOSUM62 similarity matrix, we calculated the identity among all NBS-R protein sequences to test the BLAST-P analysis. The average identity score among clusters was based on n×k protein comparisons (n and k as above). The same procedure was used to select clusters that were significantly related.

### Within-genome collinearity

An all-against-all BLASTP of the whole predicted protein data set (31063 codified by the anchored *NBS*-*R* genes) identified paralogous gene pairs if their two sequences were alignable over a length of more than 150 amino acids with an identity score higher than 30% [63]. The set of paralogs was used to detect duplicated/collinear segments by running i-ADHoRe version 2.0 [69], with the gap size set to 40 genes (the maximum distance between consecutive paralogs or anchors used to define a duplicated segment) and the *p*-value cutoff set to 0.001.

### Helitron-mediated *NBS*-*R* gene transposition

The 3′ region of single *NBS*-*R* genes was inspected (www.emboss.org) to identify inverted repeats forming a putative stem and loop structure (28-bp threshold, mismatch −1 and maxrepeat 30 bp). Also the CTAG signature following a regular expression script was searched by imposing a cut-off between CTAG and stem-loop structure [30]. Kapitonov and Jurka [30] have proposed three models of helitron transposition that differ in type and size of DNA sequences that remain *in situ*. All models accept that the stem and loop structure and the CTAG signature remain at the excision site in the 3′ of the mobilized genes.

### Time of transposition and cluster formation events

Time of transposition events was calculated from Ks values between putative progenitor genes and their putative helitron-transposed copies on the basis of the divergence time between Cleomaceae and Brassicaceae (a Ks value of 0.82 corresponds to 41 mya), as estimated by Schranz and Mitchell-Olds [3]. Among clustered genes, progenitors of putatively transposed genes were selected when having, in gene-to-gene comparisons, the lowest Ks value. Time of homogenous *NBS*-*R* cluster formation was inferred based on Ks-w values.

## Supporting Information

Figure S1 **Distribution of Ks-bg and Ks-w scores.** Ks-bg scores were calculated by comparing protein products of *NBS*-*R* genes of different clusters and of single *NBS*-*R* genes. Ks-w scores were derived from pairwise comparisons of products of *NBS*-*R* genes belonging to the same cluster.
(TIF)

Figure S2 **Relationships between grapevine chromosomes based on the 96th (A) and 90th (B) percentile of the distribution of BLAST bit scores among clusters.** BLAST bit scores were generated by a pairwise BLAST-P protein sequence comparison. Va (green) and Vc (red) define two genomes based on the chromosome groups indicated.
(TIF)

Figure S3 **Putative Va and Vc grapevine genomes.** The formation of homologous triplets is based on the homology among groups of genes located on specific chromosome fragments. Chromosomes were assigned to Va (green) and Vc (red) genomes by cluster similarity results and by identity scores.
(TIF)

Table S1 **Grapevine** ***NBS*** **-** ***R*** **genes of Pinot Noir genome sequence Release 3 (** http://genomics.research.iasma.it/ **).**
(XLS)

Table S2 **Organization and distribution of** ***NBS*** **-** ***R*** **genes in the Pinot Noir grapevine genome.**
(DOC)

Table S3 **Chromosome organization of** ***NBS*** **-** ***R*** **genes in the Pinot Noir grapevine genome.**
(DOC)

Table S4 ***NBS*** **-** ***R*** **gene cluster size and structure, and their chromosomal position, phylogenetic subclade, and similarity (BLAST bit scores higher than the 93rd percentile) with others** ***NBS*** **-** ***R*** **gene clusters.**
(DOC)

Table S5 ***NBS*** **-** ***R*** **gene clusters present in A to M phylogenetic subclades and assignment to Va and Vc genomes.**
(DOC)

Table S6 **Assignment of grapevine chromosomes to Va and Vc genomes together with** ***NBS*** **-** ***R*** **gene cluster and their phylogenetic subclades**.
(DOC)

Table S7 **Distribution of** ***NBS*** **-** ***R*** **genes based on phylogenetic subclades and on specific protein domains of clustered (CL) and single (R)** ***NBS*** **-** ***R*** **genes present on the unassigned chromosomes 4, 11, 14, 16, and 17.**
(DOC)

Table S8 **Presence in 55 single (R) and six clustered (CL)** ***NBS*** **-** ***R*** **genes of features of helitron transpositive activity, which include the CTAG tetranucleotide and the inverted repeat forming the stem and loop structure.** The table includes some of the putative ancestor genes from which single genes may have originated.
(DOC)

Text S1 **Supporting text and references.** **S1A.** Angiosperm phylogeny. **S1B.** Rosids fossils. **S1C.** The family of Vitaceae.
(DOC)
