# Supplementary figures and images for: Deconstruction of the (Paleo)Polyploid Grapevine Genome Based on the Analysis of Transposition Events Involving NBS Resistance Genes
Source: PLoS One. 2012 Jan 11;7(1):e29762. doi: 10.1371/journal.pone.0029762 (PMC3256180; doi:10.1371/journal.pone.0029762)

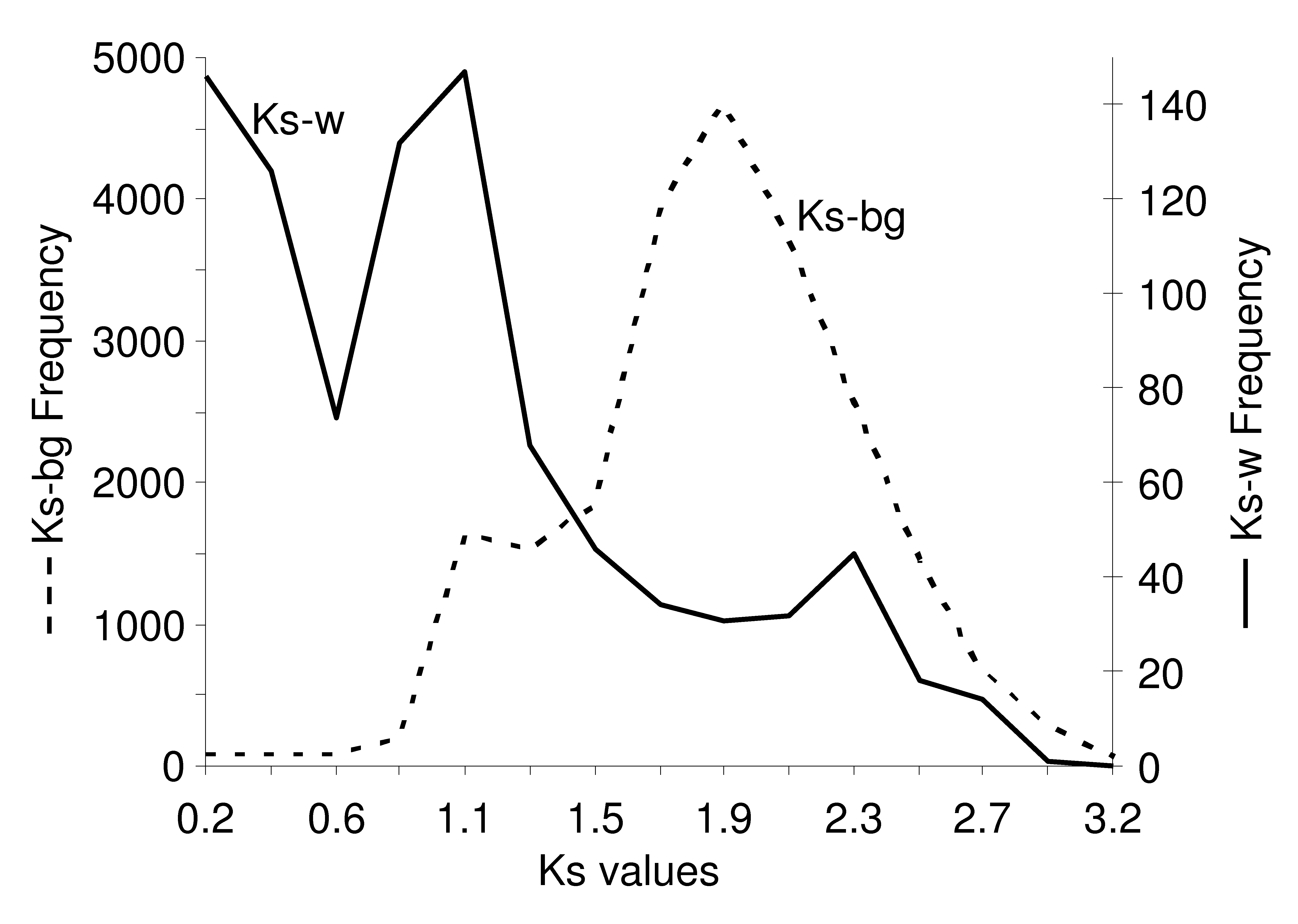

Supplement: Figure S1 — Distribution of Ks-bg and Ks-w scores. Ks-bg scores were calculated by comparing protein products of NBS-R genes of different clusters and of single NBS-R genes. Ks-w scores were derived from pairwise comparisons of products of NBS-R genes belonging to the same cluster. (TIF) [file pone.0029762.s001.tif]
