# Supplementary material for: Deconstruction of the (Paleo)Polyploid Grapevine Genome Based on the Analysis of Transposition Events Involving NBS Resistance Genes
Source: PLoS One. 2012 Jan 11;7(1):e29762. doi: 10.1371/journal.pone.0029762 (PMC3256180; doi:10.1371/journal.pone.0029762)
Supplement: Table S2 — Organization and distribution of NBS - R genes in the Pinot Noir grapevine genome. (DOC) [file pone.0029762.s005.doc]

**Table S2.** Organization and distribution of *NBS*-*R* genes in the Pinot Noir grapevine genome.

| **Chromosome** | **Total *NBS-R* genes** | **Genomic organization** | |
| --- | --- | --- | --- |
|  |  | **Clusters (Clustered *NBS-R* genes)** | **Single *NBS-R* genes** |
| 1 | 12 | 2 (9) | 3 |
| 2 | 5 | 1 (4) | 1 |
| 3 | 18 | 3 (16) | 2 |
| 4 | 1 | 0 | 1 |
| 5 | 17 | 2 (13) | 4 |
| 6 | 4 | 1 (2) | 2 |
| 7 | 21 | 4 (17) | 4 |
| 8 | 6 | 1 (4) | 2 |
| 9 | 45 | 7 (42) | 3 |
| 10 | 6 | 1 (3) | 3 |
| 11 | 9 | 2 (5) | 4 |
| 12 | 36 | 4 (35) | 1 |
| 13 | 67 | 8 (65) | 2 |
| 14 | 2 | 0 | 2 |
| 15 | 23 | 4 (20) | 3 |
| 16 | 6 | 0 | 6 |
| 17 | 3 | 1 (2) | 1 |
| 18 | 35 | 5 (29) | 6 |
| 19 | 30 | 6 (25) | 5 |
|  |  |  |  |
| not positioned | 45 |  |  |
|  |  |  |  |
| **Total** | **391** | **52 (291)** | **55** |
