# Supplementary material for: Deconstruction of the (Paleo)Polyploid Grapevine Genome Based on the Analysis of Transposition Events Involving NBS Resistance Genes
Source: PLoS One. 2012 Jan 11;7(1):e29762. doi: 10.1371/journal.pone.0029762 (PMC3256180; doi:10.1371/journal.pone.0029762)
Supplement: Table S3 — Chromosome organization of NBS - R genes in the Pinot Noir grapevine genome. (DOC) [file pone.0029762.s006.doc]

**Table S3.** Chromosome organization of *NBS*-*R* genes in the Pinot Noir grapevine genome.

| **Chr** | **Total *NBS-R* genes** | **Classification by protein domains** | | | | | | | | | | | |
| --- | --- | --- | --- | --- | --- | --- | --- | --- | --- | --- | --- | --- | --- |
|  |  | ***CC-NBS*** | | ***CC-NBS-LRR*** | | ***TIR-NBS*** | | ***TIR-NBS-LRR*** | | ***NBS-LRR*** | | ***NBS-tr*** | |
|  |  | **Clustered genes** | **Single genes** | **Clustered genes** | **Single genes** | **Clustered genes** | **Single genes** | **Clustered genes** | **Single genes** | **Clustered genes** | **Single genes** | **Clustered genes** | **Single genes** |
|  |  |  |  |  |  |  |  |  |  |  |  |  |  |
| 1 | 12 | - | - | 2 | 1 | - | - | 2 | 1 | 4 | 1 | 1 | - |
| 2 | 5 | - | - | 1 | - | - | - | - | - | 2 | 1 | 1 | - |
| 3 | 18 | 1 | 1 | 2 | 1 | - | - | - | - | 5 | - | 8 | - |
| 4 | 1 | - | - | - | 1 | - | - | - | - | - | - | - | - |
| 5 | 17 | - | 1 | - | 2 | - | - | 2 | - | 5 | 1 | 6 | - |
| 6 | 4 | - | - | - | - | - | - | - | - | 1 | 2 | 1 | - |
| 7 | 21 | 1 | - | 4 | 1 | - | - | - | - | 9 | 3 | 3 | - |
| 8 | 6 | 1 | - | 1 | - | - | - | - | - | 2 | 2 | - | - |
| 9 | 45 | 9 | - | 21 | 1 | - | - | - | 1 | 5 | 1 | 7 | - |
| 10 | 6 | - | - | 2 | - | - | 1 | - | - | 1 | 2 | - | - |
| 11 | 9 | - | - | 2 | 2 | - | - | 1 | - | 1 | 1 | 1 | 1 |
| 12 | 36 | 2 | - | 15 | 1 | - | - | 2 | - | 13 | - | 3 | - |
| 13 | 67 | 3 | - | 20 | 1 | - | - | 1 | - | 34 | 1 | 7 | - |
| 14 | 2 | - | - | - | - | - | - | - | - | - | 2 | - | - |
| 15 | 23 | - | - | 3 | - | - | - | - | 1 | 5 | - | 12 | 2 |
| 16 | 6 | - | - | - | - | - | - | - | - | - | 4 | - | 2 |
| 17 | 3 | 1 | 1 | 1 | - | - | - | - | - | - | - | - | - |
| 18 | 35 | - | - | 1 | - | 3 | - | 11 | 2 | 13 | 3 | 1 | 1 |
| 19 | 30 | 5 | - | 10 | - | - | 1 | - | - | 6 | 2 | 4 | 2 |
|  |  |  |  |  |  |  |  |  |  |  |  |  |  |
| np | 45 | 6 | | 15 | | 1 | | 3 | | 13 | | 7 | |
|  |  |  |  |  |  |  |  |  |  |  |  |  |  |
| **Total** | **391** | **32** | | **111** | | **6** | | **27** | | **145** | | **70** | |

np: not positioned on grapevine chromosomes.
