# Supplementary material for: Deconstruction of the (Paleo)Polyploid Grapevine Genome Based on the Analysis of Transposition Events Involving NBS Resistance Genes
Source: PLoS One. 2012 Jan 11;7(1):e29762. doi: 10.1371/journal.pone.0029762 (PMC3256180; doi:10.1371/journal.pone.0029762)
Supplement: Table S4 — NBS - R gene cluster size and structure, and their chromosomal position, phylogenetic subclade, and similarity (BLAST bit scores higher than the 93rd percentile) with others NBS - R gene clusters. (DOC) [file pone.0029762.s007.doc]

**Table S4.** *NBS*-*R* gene cluster size and structure, and their chromosomal position, phylogenetic subclade, and similarity (BLAST bit scores higher than the 93rd percentile) with others *NBS*-*R* gene clusters.

| **Cluster** | **Chr** | **Cluster size (Kb)** |  | **Gene number** | **Subclade code^1^** |  | **Significant similar clusters^2^** |
| --- | --- | --- | --- | --- | --- | --- | --- |
| CL1 | 1 | 165,3 |  | 7 | E (6), ε(1) |  | - |
| CL2 | 1 | 32,6 |  | 2 | A(2) |  | CL7 (1366); CL46 (1418) |
| CL3 | 2 | 485,0 |  | 4 | F(1), I(3) |  | CL5 (1714); CL31 (1517); CL32 (1628); CL33 (1399); CL34 (1434); CL35 (1773) |
| CL4 | 3 | 218,6 |  | 11 | G(11) |  | CL29 (1373) |
| CL5 | 3 | 19,7 |  | 2 | *uc*(2) |  | CL3 (1714); CL13 (1533); CL25 (1343); CL30 (1392); CL31 (2001); CL32 (2078); CL33 (1795); CL34 (1689); CL35 (1739); CL36 (1460); CL44 (1651) |
| CL6 | 3 | 55,1 |  | 3 | H(3) |  | CL8 (3536); CL9 (3273) |
| CL7 | 5 | 54,9 |  | 3 | A(2), *uc*(1) |  | CL2 (1366); CL42 (1488); CL45 (1589); CL46 (1685) |
| CL8 | 5 | 201,9 |  | 10 | H(10) |  | CL6 (3536); CL9 (3298) |
| CL9 | 6 | 47,0 |  | 2 | H(2) |  | CL6 (3273); CL8 (3298) |
| CL10 | 7 | 495,5 |  | 7 | E(6), γ(1) |  | - |
| CL11 | 7 | 74,5 |  | 4 | B(4) |  | - |
| CL12 | 7 | 244,5 |  | 3 | B(1), γ(2) |  | - |
| CL13 | 7 | 20,6 |  | 3 | M(3) |  | CL5 (1533); CL25 (1588); CL26 (2446); CL27 (1997); CL30 (1741); CL31 (1481); CL32 (1612); CL33 (1626); CL36 (2276) |
| CL14 | 8 | 44,4 |  | 4 | C(3), J(1) |  | CL18 (1374); CL22 (1821); CL38 (1981) |
| CL15 | 9 | 24,1 |  | 2 | C(2) |  | CL16 (2269); CL17 (1705); CL18 (1816); CL22 (1555); CL51 (1837); CL52 (1504) |
| CL16 | 9 | 518,1 |  | 6 | C(6) |  | CL15 (2269); CL17 (1754); CL18 (1932); CL22 (1637); CL51 (1899); CL52 (1506) |
| CL17 | 9 | 189,2 |  | 10 | C(10) |  | CL15 (1705); CL16 (1754); CL18 (1844); CL22 (1438); CL51 (1536) |
| CL18 | 9 | 310,9 |  | 5 | C(5) |  | CL14 (1374); CL15 (1816); CL16 (1932); CL17 (1844); CL22 (1939); CL38 (1937); CL51 (1730); CL52 (1401) |
| CL19 | 9 | 483,7 |  | 15 | J(15) |  | - |
| CL20 | 9 | 47,2 |  | 2 | J(1), γ(1) |  | - |
| CL21 | 9 | 4,2 |  | 2 | F(2) |  | CL39 (1948); CL40 (1664) |
| CL22 | 10 | 71,0 |  | 3 | C(3) |  | CL14 (1821); CL15 (1855); CL16 (1637); CL17 (1438); CL18 (1939); CL38 (2003); CL51 (1463) |
| CL23 | 11 | 428,0 |  | 2 | A(1), C(1) |  | - |
| CL24 | 11 | 100,7 |  | 3 | γ(3) |  | - |
| CL25 | 12 | 564,6 |  | 9 | A(2), M(7) |  | CL5 (1343); CL13 (1588); CL26 (1526); CL27 (1433); CL30 (1358); CL32 (1385); CL33 (1370); CL36 (1573) |
| CL26 | 12 | 534,9 |  | 7 | M(6), γ(1) |  | CL13 (2446); CL25 (1526); CL27 (1491); CL30 (1464); CL32 (1333); CL33 (1367); CL36 (1935) |
| CL27 | 12 | 554,6 |  | 7 | M(7) |  | CL13 (1797); CL25 (1433); CL26 (1491); CL30 (1360); CL36 (1792) |
| CL28 | 12 | 475,3 |  | 12 | D(8), α(3), β(1) |  | - |
| CL29 | 13 | 96,6 |  | 4 | G(4) |  | CL4 (1373) |
| CL30 | 13 | 331,0 |  | 6 | A(1), I(1), M(4) |  | CL5 (1392); CL13 (1741); CL25 (1358); CL26 (1464); CL27 (1360); CL32 (1367); CL33 (1341); CL36 (2336) |
| CL31 | 13 | 106,4 |  | 3 | K(1), M(2) |  | CL3 (1517); CL5 (2001); CL13 (1481); CL32 (2270); CL33 (1727); CL34 (1509); CL35 (1840); CL36 (1396); CL44 (1412) |
| CL32 | 13 | 240,0 |  | 15 | M(15) |  | CL3 (1628); CL5 (2078); CL13 (1612); CL25 (1385); CL26 (1333); CL30 (1367); CL31 (2270); CL33 (1943); CL34 (1595); CL35 (1659); CL36 (1524); CL44 (1457) |
| CL33 | 13 | 544,7 |  | 12 | M(12) |  | CL3 (1399); CL5 (1795); CL13 (1626); CL25 (1370); CL26 (1367); CL30 (1341); CL31 (1727); CL32 (1943); CL34 (1636); CL35 (1503); CL36 (1597) |
| CL34 | 13 | 422,4 |  | 10 | I(6), M(2), C(2) |  | CL3 (1434); CL5 (1689); CL31 (1509); CL32 (1595); CL33 (1636); CL35 (1542) |
| CL35 | 13 | 742,1 |  | 10 | I(6), K(3), *uc*(1) |  | CL3 (1773); CL5 (1739); CL31 (1840); CL32 (1659); CL33 (1503); CL34 (1542) |
| CL36 | 13 | 127,1 |  | 5 | M(5) |  | CL5 (1460); CL13 (2276); CL25 (1573); CL26 (1935); CL27 (1792); CL30 (2336); CL31 (1396); CL32 (1524); CL33 (1597) |
| CL37 | 15 | 226,2 |  | 5 | ε(2), ζ(3) |  | - |
| CL38 | 15 | 103,1 |  | 3 | C(3) |  | CL14 (1981); CL18 (1397); CL22 (2003) |
| CL39 | 15 | 16,9 |  | 2 | F(2) |  | CL21 (1948); CL40 (1684) |
| CL40 | 15 | 611,9 |  | 10 | F(9), G(1) |  | CL21 (1664); CL39 (1684) |
| CL41 | 17 | 3,6 |  | 2 | ε(2) |  | - |
| CL42 | 18 | 584,3 |  | 4 | A(4) |  | CL7 (1488); CL45 (1330); CL46 (1576) |
| CL43 | 18 | 406,7 |  | 8 | A(6), I(1), M(1) |  | - |
| CL44 | 18 | 400,7 |  | 7 | A(3), I(4) |  | CL5 (1651); CL31 (1412); CL32 (1457) |
| CL45 | 18 | 126,9 |  | 8 | A(8) |  | CL7 (1589); CL42 (1330); CL46 (1400); |
| CL46 | 18 | 50,3 |  | 2 | A(2) |  | CL2 (1418); CL7 (1685); CL42 (1576); CL45 (1400) |
| CL47 | 19 | 36,6 |  | 4 | γ(3), δ(1) |  | - |
| CL48 | 19 | 32,7 |  | 2 | E(2) |  | - |
| CL49 | 19 | 280,8 |  | 3 | C(1), K81), L(1) |  | - |
| CL50 | 19 | 328,2 |  | 4 | L(3), ε(1) |  | - |
| CL51 | 19 | 82,9 |  | 5 | C(5) |  | CL15 (1837); CL16 (1899); CL17 (1593) CL18 (1730); CL22 (1463); CL52 (1682) |
| CL52 | 19 | 232,1 |  | 7 | C(5), L(2) |  | CL15 (1504); CL16 (1506); CL18 (1401); CL51 (1682) |

^1^ Numbers of genes for each clade are given in brackets.

^2^ Significant similar clusters with a cluster similarity scores higher than the 93rd percentile BLAST bit scores are reported in brackets.

uc (unclassified): genes not assigned to phylogenetic subclades.
