# Supplementary material for: Deconstruction of the (Paleo)Polyploid Grapevine Genome Based on the Analysis of Transposition Events Involving NBS Resistance Genes
Source: PLoS One. 2012 Jan 11;7(1):e29762. doi: 10.1371/journal.pone.0029762 (PMC3256180; doi:10.1371/journal.pone.0029762)
Supplement: Table S5 — NBS - R gene clusters present in A to M phylogenetic subclades and assignment to Va and Vc genomes. (DOC) [file pone.0029762.s008.doc]

**Table S5.** *NBS*-*R* gene clusters present in A to M phylogenetic subclades and assignment to Va and Vc genomes.

| **Subclade** | **Cluster** | **Chromosome** | | |
| --- | --- | --- | --- | --- |
|  |  | **Va genome** | **Vc genome** | **Unassigned** |
| A | CL2, CL7, CL23, CL25, CL30, CL42, CL43, CL44, CL45, CL46 | 1, 5, 12, 13, 18 |  | 11 |
| B | CL11, CL12 | 7 |  |  |
| C | CL14, CL15, CL16, CL17, CL18, CL22, CL23, CL38, CL49, CL51, CL52 |  | 8, 9, 10, 15, 19 | 11 |
| D | CL28 | 12 |  |  |
| E | CL1, CL10, CL48 | 1, 7 | 19 |  |
| F | CL3, CL21, CL39, CL40 | 2 | 9, 15 |  |
| G | CL4, CL29, CL40 | 3,13 | 15 |  |
| H | CL6, CL8, CL9 | 3, 5, 6 |  |  |
| I | CL3, CL30, CL34, CL35, CL43, CL44 | 2, 13, 18 |  |  |
| J | CL14, CL19, CL20 |  | 8, 9 |  |
| K | CL31, CL35, CL49 | 13 | 19 |  |
| L | CL49, CL50, CL52 |  | 19 |  |
| M | CL13, CL25, CL26, CL27, CL30, CL31, CL32, CL33, CL34, CL36, CL43 | 7, 12, 13, 18 |  |  |

Unassigned: chromosomes not assigned to Va or Vc genomes.
