# Supplementary material for: Deconstruction of the (Paleo)Polyploid Grapevine Genome Based on the Analysis of Transposition Events Involving NBS Resistance Genes
Source: PLoS One. 2012 Jan 11;7(1):e29762. doi: 10.1371/journal.pone.0029762 (PMC3256180; doi:10.1371/journal.pone.0029762)
Supplement: Table S6 — Assignment of grapevine chromosomes to Va and Vc genomes together with NBS - R gene cluster and their phylogenetic subclades. (DOC) [file pone.0029762.s009.doc]

**Table S6.** Assignment of grapevine chromosomes to Va and Vc genomes together with *NBS*-*R* gene cluster and their phylogenetic subclades*.*

| **Chromosome** | **Genome** | **Cluster** | **Subclade of clustered genes** | |
| --- | --- | --- | --- | --- |
| 1 | Va | CL1, CL2 | A, E | ε |
| 2 | Va | CL3 | F, I |  |
| 3 | Vb | CL4, CL5, CL6 | G, H |  |
| 4 | Va | - |  |  |
| 5 | Va | CL7, CL8 | A, H |  |
| 6 | Vb | CL9 | H |  |
| 7 | Vb | CL10, CL11, CL12, CL13 | B, E, M | γ |
| 8 | Vc | CL14 | C, J |  |
| 9 | Vc | CL15, CL16, CL17, CL18, CL19, CL20, CL21 | C, F, J | γ |
| 10 | Vc | CL22 | C |  |
| 11 | Vb | CL23, CL24 | A, C | γ |
| 12 | Va | CL25, CL26, CL27, CL28 | A, D, M | α, β, γ |
| 13 | Va | CL29, CL30, CL31, CL32, CL33, CL34, CL35, CL36 | A, G, I, K, M |  |
| 14 | Vc | - |  |  |
| 15 | Vc | CL37, CL38, CL39, CL40 | C, F, G | ε, ζ |
| 16 | Vb | - |  |  |
| 17 | Vb | CL41 |  | ε |
| 18 | Va | CL42, CL43, CL44, CL45, CL46 | A, I, M |  |
| 19 | Vc | CL47, CL48, CL49, CL50, CL51, CL52 | C, E, K, L | ε, δ, γ |
