# Supplementary material for: Deconstruction of the (Paleo)Polyploid Grapevine Genome Based on the Analysis of Transposition Events Involving NBS Resistance Genes
Source: PLoS One. 2012 Jan 11;7(1):e29762. doi: 10.1371/journal.pone.0029762 (PMC3256180; doi:10.1371/journal.pone.0029762)
Supplement: Table S7 — Distribution of NBS - R genes based on phylogenetic subclades and on specific protein domains of clustered (CL) and single (R) NBS - R genes present on the unassigned chromosomes 4, 11, 14, 16, and 17. (DOC) [file pone.0029762.s010.doc]

**Table S7.** Distribution of *NBS*-*R* genes based on phylogenetic subclades and on specific protein domains of clustered (CL) and single (R) *NBS*-*R* genes present on the unassigned chromosomes 4, 11, 14, 16, and 17.

| ***NBS* subclade** |  | ***NBS* class** | | | | | | | | | | | | | |
| --- | --- | --- | --- | --- | --- | --- | --- | --- | --- | --- | --- | --- | --- | --- | --- |
|  |  | **Chr4** |  | **Chr 11** | | | |  | **Chr 14** |  | **Chr 16** | |  | **Chr 17** | |
|  |  | ***CC-NBS-LRR*** |  | ***CC-NBS-LRR*** | ***TIR-NBS-LRR*** | ***NBS-LRR*** | ***NBS-tr*** |  | ***NBS-LRR*** |  | ***NBS-LRR*** | ***NBS-tr*** |  | ***CC-NBS-LRR*** | ***CC-NBS*** |
| A | CL | - |  | - | 1 | - | - |  | - |  | - | - |  | - | - |
|  | R | - |  | - | - | - | - |  | - |  | - | - |  | - | - |
| B | CL | - |  | - | - | - | - |  | - |  | - | - |  | - | - |
|  | R | - |  | - | - | - | - |  | - |  | - | - |  | - | - |
| C | CL | - |  | - | - | - | 1 |  | - |  | - | - |  | - | - |
|  | R | - |  | 2 | - | - | - |  | - |  | - | - |  | - | 1 |
| D | CL | - |  | - | - | - | - |  | - |  | - | - |  | - | - |
|  | R | - |  | - | - | - | - |  | - |  | - | - |  | - | - |
| E | CL | - |  | - | - | - | - |  | - |  | - | - |  | - | - |
|  | R | 1 |  | - | - | - | - |  | - |  | - | - |  | - | - |
| F | CL | - |  | - | - | - | - |  | - |  | - | - |  | - | - |
|  | R | - |  | - | - | - | - |  | - |  | - | 2 |  | - | - |
| G | CL | - |  | - | - | - | - |  | - |  | - | - |  | - | - |
|  | R | - |  | - | - | - | 1 |  | - |  | - | - |  | - | - |
| H | CL | - |  | - | - | - | - |  | - |  | - | - |  | - | - |
|  | R | - |  | - | - | - | - |  | - |  | - | - |  | - | - |
| I | CL | - |  | - | - | - | - |  | - |  | - | - |  | - | - |
|  | R | - |  | - | - | - | - |  | - |  | 2 | - |  | - | - |
| J | CL | - |  | - | - | - | - |  | - |  | - | - |  | - | - |
|  | R | - |  | - | - | - | - |  | - |  | - | - |  | - | - |
| K | CL | - |  | - | - | - | - |  | - |  | - | - |  | - | - |
|  | R | - |  | - | - | - | - |  | - |  | - | - |  | - | - |
| L | CL | - |  | - | - | - | - |  | - |  | - | - |  | - | - |
|  | R | - |  | - | - | - | - |  | - |  | - | - |  | - | - |
| M | CL | - |  | - | - | - | - |  | - |  | - | - |  | - | - |
|  | R | - |  | - | - | - | - |  | - |  | - | - |  | - | - |
| not assigned to A-M | CL | - |  | 2 | - | 1 | - |  | - |  | - | - |  | 1 | 1 |
|  | R | - |  | - | - | 1 | - |  | 2 |  | 2 | - |  | - | - |
