# Supplementary material for: Deconstruction of the (Paleo)Polyploid Grapevine Genome Based on the Analysis of Transposition Events Involving NBS Resistance Genes
Source: PLoS One. 2012 Jan 11;7(1):e29762. doi: 10.1371/journal.pone.0029762 (PMC3256180; doi:10.1371/journal.pone.0029762)
Supplement: Table S8 — Presence in 55 single (R) and six clustered (CL) NBS - R genes of features of helitron transpositive activity, which include the CTAG tetranucleotide and the inverted repeat forming the stem and loop structure. The table includes some of the putative ancestor genes from which single genes may have originated. (DOC) [file pone.0029762.s011.doc]

**Table S8.** Presence in 55 single (R) and six clustered (CL) *NBS*-*R* genes of features of helitron transpositive activity, which include the CTAG tetranucleotide and the inverted repeat forming the stem and loop structure. The table includes some of the putative ancestor genes from which single genes may have originated.

| ***NBS* genes** | **Subclade** | **Genome** | **Nucleotides from STOP codon** | **Bp^1^** |
| --- | --- | --- | --- | --- |
| *R297* | A | Va | 1371 | 21 |
| *R284* | A | Va | 1720 | 17 |
| *R10* | A | Va | - | - |
| *R316* | A | Va | - | - |
| *R125* | A | Vc | 446 | 9 |
| *R132* | A | Vc | 283 | 9 |
| *R255* | A | Vc | 985 | 9 |
| *R321* | A | Vc | - | - |
| *R323* | A | Vc | - | - |
| *R43* | C | Va | - | - |
| *R126* | C | Vc | - | - |
| *R136* | C | Vb | - | - |
| *R141* | C | na | - | - |
| *R281* | C | na | - | - |
| *R38* | E | Va | n.d. | n.d. |
| *R54* | E | Va | n.d. | n.d. |
| *R145* | E | Va | - | - |
| *R131* | E | Vc | - | - |
| *R79* | E | Vc | - | - |
| *R36* | E | Va | - | - |
| *R13* | epsilon | Va | - | - |
| *R276* | epsilon | na | n.d. | n.d. |
| *R277* | epsilon | na | - | - |
| *R257* | F | Vc | n.d. | n.d. |
| *R273* | F | na | - | - |
| *R274* | F | na | - | - |
| *R256* | G | Vc | 471 | 18 |
| *R130* | G | Vc | - | - |
| *R137* | G | na | 2015 | 20 |
| *R283* | gamma | Va | - | - |
| *R322* | gamma | Vc | - | - |
| *R248* | gamma | Vc | 2733 | 23 |
| *R249* | gamma | Vc | - | - |
| *R138* | gamma | na | - | - |
| *R11* | I | Va | - | - |
| *R37* | I | Va | - | - |
| *R185* | I | Va | - | - |
| *R186* | I | Va | - | - |
| *R346* | I | Vc | 2336 | 18 |
| *R275* | I | na | - | - |
| *R278* | I | na | 72 | 13 |
| *R29* | J | Va | 2091 | 12 |
| *R39* | J | Va | 344 | 14 |
| *R58* | J | Va | - | - |
| *R345* | L | Vc | - | - |
| *R55* | M | Va | 934 | 4 |
| *R67* | M | Va | - | - |
| *R68* | M | Va | - | - |
| *R282* | M | Va | - | - |
| *R129* | M | Vc | 201 | 18 |
| *R313* | unclassified | Va | - | - |
| *R12* | unclassified | Va | - | - |
| *R30* | unclassified | Va | - | - |
| *R59* | unclassified | Va | - | - |
| *R84* | unclassified | Vc | 528 | 24 |
| *CL46_314* | A | Va | - | - |
| *CL16_90* | C | Vc | - | - |
| *CL4_22* | G | Va | - | - |
| *CL35_237* | I | Va | - | - |
| *CL19_113* | J | Vc | - | - |
| *CL32_208* | M | Va | - | - |

^1^ Number of bp between the putative stem and loop structure and the CTAG tetranucleotide.

nd: not detectable due to the length of the contig.
