# Supplementary material for: Deconstruction of the (Paleo)Polyploid Grapevine Genome Based on the Analysis of Transposition Events Involving NBS Resistance Genes
Source: PLoS One. 2012 Jan 11;7(1):e29762. doi: 10.1371/journal.pone.0029762 (PMC3256180; doi:10.1371/journal.pone.0029762)
Supplement: Text S1 — Supporting text and references. S1A. Angiosperm phylogeny. S1B. Rosids fossils. S1C. The family of Vitaceae. (DOC) [file pone.0029762.s012.doc]

**Supporting Text**

**S1A. Angiosperm phylogeny (from Friis et al. [1])**

Molecular analyses have generated models of angiosperm phylogeny that elucidates the relationships among extant orders [2-4]. At the same time, palaeobotanical and palynological studies of Cretaceous angiosperms have contributed to date the major evolutionary events in angiosperm history [5-15]. The integration of the two types of data offers new insights into diversification rate and age of different angiosperm clades [16-22]. The fossil record supports the presence of diverse angiosperms in the Early Cretaceous, and indicates that many major lineages of extant taxa differentiated by the early Late Cretaceous, with a distinctive and regular expansion in diversity through the mid- to late-Early Cretaceous (Hauterivian–Barremian–Aptian–Albian). The high diversity in the fossil record in the Cretaceous has raised questions about possible pre-Cretaceous precursors. Most analyses of divergence time for angiosperms using molecular techniques have concluded that their ages are older than those of the fossil record [21]. However, there are no definite evidence of pre-Cretaceous angiosperms.

A new line of development considers rates of molecular divergence to estimate the time of deep splits between angiosperm lineages. The target is to convert genetic distances into times based on ‘molecular clock’ assumptions [9,16-21,23-43], although strikingly different rates of molecular evolution are reported for different angiosperm lineages [44]. Results are confusing and time estimation of the same divergence can vary by tens of or even hundreds of millions of years by using different approaches.

**Examples of the difficulty to assemble different sets of data**.

Morphological resemblance between the pollen of *Asteropollis* and that of extant *Hedyosmum* (Chloranthaceae) has been documented [45]. This link has been supported by the finding of pollen grains of the *Asteropollis* type inside Cretaceous flowers with floral features of extant *Hedyosmum* [46,47]. On the basis of molecular data, a mid-Cenozoic age (45 mya) was suggested for the initial divergence of extant *Hedyosmum* species [20]. Eklund [34] showed that the Cretaceous fossils fit equally well below, within or above the three basal species of extant *Hedyosmum* (Figure 1). Taking into account the proposed late divergence of the crown group *Hedyosmum*, which include all extant and extinct species that diverged after the origin of the most recent common ancestor, it has been argued that for the fossils is more likely a stem-group position. This evidence implies that not all the features (synapomorphies) that define the genus were present in the fossils and can indicates that *Hedyosmum* experienced two major phases of diversification: the first phase, an Early Cretaceous radiation followed by Late Cretaceous extinctions; and the second phase, mid-Cenozoic radiation that generated the extant diversity. Alternative explanations refer to the possibility that the age calculated for the crown group is erroneous. A comparable apparent conflict between molecular ages and the fossil record has been demonstrated for the genus *Ephedra* of the Gnetales [48].

**Figure 1 from Eklund [34].**

Two alternative scenarios for the age of crown-group *Hedyosmum* (Chloranthaceae). (**a**) Plants with all the features typical of modern *Hedyosmum* (crown-group *Hedyosmum*) are old and they originated soon after the lineage divergence between *Hedyosmum* and its sister group. According to this scenario, modern *Hedyosmum* developed soon after the first appearance of angiosperms in the fossil record and has retained its own features for more than 120 million years. Tthis scenario is supported by the finding of fossil pollen (*Asteropollis*) and flowers indistinguishable from those of modern *Hedyosmum* [34,47]. (**b**) Crown-group *Hedyosmum* is young and originated in the Cenozoic more than 100 million years after the divergence between *Hedyosmum* and its sister group, and almost as long after the appearance of plants with distinctive *Hedyosmum* reproductive features. According to this second scenario, some of the features typical of extant *Hedyosmum* originated some time during the Late Cretaceous or Cenozoic. This hypothesis is supported by molecular dating [20], while fossil data, according to Eklund [34], are equivocal.

**S1B. Rosids fossils**

The combination of time estimations, calibrated by two known fossil models, showed that monocots branched off from dicots 140-150 mya, and that core eudicots diverged 110-115 mya (Albian-Aptian of the Cretaceous) [23]. Based on available phylogenetic evidences on dicots evolutionary trends, Friis et al. [49] consider that Magnoliides *sensu stricto*, monocots and eudicots should have shared a common ancestor with Ranunculales sister of the remainder eudicots. Vitaceae are sister of the rosids, thus their position has to be considered near to the polycotomy of core eudicots (Berberidopsidales, Santanales, Saxifragales, Rosids, Asterids, Caryophyllales and Dillaniaceae).

Based on molecular data, a rapid diversification of eudicots has been inferred at the end of the Albian [17,39,50]. However, the fossil approach indicates that the earliest evidence of eudicots is provided by tricolpate pollen grains recorded from the late Barremian-early Aptian around 120 mya [51]. In the Turonian (95 mya) [52,53], fossils from rosids and asterids are present (two flowers from the Turonian of New Jersey [33,54]), while *Platydiscus peltatus* [36] and species of *Esgueiria* [55] are more recent (85-90 mya).

**S1C. The family of Vitaceae**

The genus *Vitis* is monophyletic and forms a larger clade with Ampelocissus, Pterisanthes and Nothocissus of the Vitaceae. Vitaceae have 14 genera [56]. Leea is placed either in the Vitaceae [3,57] but also as subfamily of Vitaceae [58,59] or in the family Leaceae [60].

Soejima and Wen [56] defined 12 clades [23,49,56,61,62] that includes species of the 12 genera of Vitaceae (with *Leea* and *Dillenia* considered as outgroups).

| **Family** | **Chromosome number (n)** | **Reference** |
| --- | --- | --- |
| **Vitaceae** |  |  |
| *Ampelopsis* | 20 | [63] |
| *Ampelocissus* | 20, 40 (*A. araneosa)* | [63,64] |
| *Rhoicissus* | 20 | [63] |
| *Pterisanthes* |  |  |
| *Nothocissus* |  |  |
| *Parthenocissus* | 20 | [63] |
| *Yua* | 20 | [65] |
| *Tetrastigma* | 11 (occasionally 22, 26) | [63,64] |
| *Cayratia* | 30-40 | [66] |
| *Cyphostemma* | 10, 11, 20, 22 | [63,64] |
| *Vitis* | 19 (20) | [67] |
| *Cissus* | 12 (occasionally 11 or 13) 24,40 | [66] |
| *Landukia* | 20 | [63] |
| *Clematicissus* | 20 | [63] |
| **Leeaceae** |  |  |
| *Leea* | 12, 24 | [64] |
| **Celastraceae** |  |  |
| *Euonymus* | 8 (*E. echinatus*), 16 (*E. radicans*), 24 (*E. bullatus*), 32 (*E. europaeus*) | [64] |
| *Tripterygium* | 12 | [68] |
| **Rhanmaceae** |  |  |
| *Phylica* |  |  |
| *Rhamnus* | 10, 11, 13 | [64] |
| *Ziziphus* | 12 | [69] |
| **Dilleniaceae** |  |  |
| *Dillenia* | 13, 16, 24, 27 | [64] |

**Supplementary references**

1. Friis EM, Pedersen KR, Crane PR (2005) When Earth started blooming: insights from the fossil record. Curr Opin Plant Biol 8: 5-12.

2. Davies TJ, Barraclough TG, Chase MW, Soltis PS, Soltis DE, et al. (2004) Darwin's abominable mystery: Insights from a supertree of the angiosperms. Proc Natl Acad Sci U S A 101: 1904-1909.

3. APG I (2003) An update of the Angiosperm phylogeny group classification for orders and families of flowering plants: APG II. Bot J Linn Soc 141: 399-436.

4. Soltis DE, Soltis PS, Zanis MJ (2002) Phylogeny of seed plants based on evidence from eight genes. Am J Bot 89: 1670-1681.

5. Raynolds RG, Johnson KR (2003) Synopsis of the stratigraphy and paleontology of the uppermost Cretaceous and lower Tertiary strata in the Denver Basin, Colorado. Rocky Mountain Geology 38: 171-181.

6. Friis EM, Doyle JA, Endress PK, Leng Q (2003) Archaefructus-angiosperm precursor or specialized early angiosperm? Trends Plant Sci 8: 369-373.

7. Sun G, Ji Q, Dilcher DL, Zheng S, Nixon KC, et al. (2002) Archaefructaceae, a new basal angiosperm family. Science 296: 899-904.

8. Dilcher DL (2001) Paleobotany: some aspects of non-flowering and flowering plant evolution. Taxon 50: 697-711.

9. Friis EM, Pedersen, K. R. & Crane, P. R. 2001 (2001) Origin and radiation of angiosperms. In: Briggs DEGC, P. R., editor. Palaeobiology II. Oxford: Blackwell Science. pp. 97-102.

10. Friis EM, Pedersen KR, Crane PR (2000) Fossil floral structures of a basal angiosperm with monocolpate, reticulate-acolumellate pollen from the Early Cretaceous of Portugal. Grana 39: 226–245.

11. Crepet WL (2000) Progress in understanding angiosperm history, success, and relationships: Darwin’s abominably ‘perplexing phenomenon’. Proc Natl Acad Sci U S A 97: 12939–12941.

12. Crepet WL (1996) Timing in the evolution of derived floral characters: Upper Cretaceous (Turonian) taxa with tricolpate and tricolpatederived pollen. Rev Palaeobot Palynol 90: 339–359.

13. Crane PR, Friis EM, Pederson KR (1995) The origin and early diversification of angiosperms. Nature 374: 27-33.

14. Doyle JA, Hickey LJ (1976) In: Beck CB, editor. Origin and early evolution of Angiosperms. New York: Columbia University Press. pp. 139-206.

15. Brenner GJ (1963) The spores and pollen of the Potomac group of Maryland. Bull Maryland Dept Geol Mines Water Resources 27: 1-215.

16. Magallón S (2004) Dating lineages: molecular and paleonotological approaches to the temporal framework of clades. Int J Plant Sci 165: S7–S21.

17. Bremer K, Fries EM, Bremer B (2004) Molecular phylogenetic dating of asterid flowering plants shows early Cretaceous diversification. Syst Biol 53: 496–505.

18. Renner SS, Zhang L-B (2004) Biogeography of the *Pistia clade* (Araceae): based on chloroplast and mitochondrial DNA sequences and Bayesian divergence time inference. Syst Biol 53: 422-432.

19. Renner SS, Zhang L-B, Murata J (2004) A chloroplast phylogeny of Arisaema (Araceae) illustrates Tertiary floristic links between Asia, North America, and East Africa. Am J Bot 91: 881-888.

20. Zhang L-B, Renner SS (2003) The deepest splits in Chloranthaceae as resolved by chloroplast sequences. Int J Plant Sci 164: S383–S392.

21. Sanderson MJ, Doyle JA (2001) Sources of error and confidence intervals in estimating the age of angiosperms from rbcL and 18S rDNA data. Am J Bot 88: 1499-1516.

22. Magallón S, Sanderson MJ (2001) Absolute diversifi cation rates in angiosperm clades. Evolution 55: 1762–1780.

23. Chaw SM, Chang CC, Chen HL, Li WH (2004) Dating the monocot-dicot divergence and the origin of core eudicots using whole chloroplast genomes. J Mol Evol 58: 424-441.

24. Friis EM, Pedersen KR, Crane PR (2004) Araceae from the Early Cretaceous of Portugal: evidence on the emergence of monocotyledons. Proc Natl Acad Sci U S A 101: 16565-16570.

25. Gandolfo MA, Nixon KC, Crepet WL (2004) Cretaceous flowers of Nymphaeaceae and implications for complex insect entrapment pollination mechanisms in early Angiosperms. Proc Natl Acad Sci U S A 101: 8056-8060.

26. Kvacek J, Eklund H (2003) A report on newly recovered reproductive structures from the Cenomanian of Bohemia (Central Europe). Int J Plant Sci 194: 1021-1039.

27. Hermsen EJ, Gandolfo MA, Nixon KC, Crepet WL (2003) Divisestylus gen. nov. (aff. Iteaceae), a fossil saxifrage from the Late Cretaceous of New Jersey, USA. Am J Bot 90: 1373-1388.

28. Leng Q, Friis E-M (2003) Sinocarpus decussa-tus gen. et sp. nov., a new angiosperm with basally syncarpous fruits from the Yixian Formation of Northeast China. Plant Syst Evol 241: 77-88.

29. Maslova N, Kodrul T (2003) New platanaceous inflorescence Archaranthus gen. nov. from the Maastrichtian-Paleocene of the Amur Region. Paleontological J 37: 89-98.

30. Mohr BAR, Ecklund H (2003) Araripia florifera, a magnoliid angiosperm from the Lower Cretaceous Crato formation (Brazil). Rev Palaeobot Palynol 126: 279-292.

31. Kennedy EM, Lovis JD, Daniel IL (2003) Discovery of a Cretaceous angiosperm reproductive structure from New Zealand. N Z J Sci Technol Sect B 46: 519-522.

32. Takahashi M, Crane PR, Manchester SR (2002) Hironoia fusiformis gen. et sp. nov.: A cornalean fruit from the Kamikitaba locality (Upper Cretaceous, Lower Coniacian) in northeastern Japan. J Plant Research 115: 463-473.

33. Gandolfo MA, Nixon KC, Crepet WL (2002) Triuridaceae fossil flowers from the Upper Cretaceous of New Jersey. Am J Bot 89: 1940-1957.

34. Eklund H (2003) First Cretaceous flowers from Antarctica. Rev Palaeobot Palynol 127: 187-217.

35. Schonenberger J, Friis EM (2001) Fossil flowers of ericalean affinity from the Late Cretaceous of southern Sweden. Am J Bot 88: 467–480.

36. Schonenberger J, Friis EM, Matthews ML, Endress PK (2001) Cunoniaceae in the Cretaceous of Europe: evidence from fossil flowers. Ann Bot 88: 423-437.

37. Schonenberger J, Pedersen KR, Friis EM (2001) Normapolles flowers of fagalean affinity from the Late Cretaceous of Portugal. Pl Syst Evol 226: 205-230.

38. Wikstrom N, Savolainen V, Chase MW (2001) Evolution of the angiosperms: calibrating the family tree. Proc Biol Sci 268: 2211-2220.

39. Bremer K (2000) Early Cretaceous lineages of monocot flowering plants. Proc Natl Acad Sci U S A 97: 4707-4711.

40. Chaw SM, Parkinson CL, Cheng Y, Vincent TM, Palmer JD (2000) Seed plant phylogeny inferred from all three plant genomes: monophyly of extant gymnosperms and the origin of Gnetales from conifers. Proc Natl Acad Sci U S A 97: 4086–4091.

41. Eklund H (2000) Lauraceous flowers from the Late Cretaceous of North Carolina, USA. Bot J Linn Soc 132: 397-428.

42. Renner SS, Foreman DB, Murray D (2000) Timing transantarctic dispersal in the Antherospermataceae (Laurales):evidence from coding and noncoding chloroplast sequences. Syst Biol 49: 579-591.

43. Herendeen PS, Magallón-Puebla S, Lupia R, Crane PR, Kobylinskya J (1999) A preliminary conspectus of the Allon flora from the Late Cretaceous (late Santonian) of central Georgia, USA. Ann Mo Bot Gard 86: 407-471.

44. Sanderson MJ (2002) Estimating absolute rates of molecular evolution and divergence times: a penalized likelihood approach. Mol Biol Evol 19: 101-109.

45. Walker JW, Walker AG (1984) Ultrastructure of Lower Cretaceous Angiosperm pollen and the origin and early evolution of flowering plants. Ann Mo Bot Gard 71: 464-521.

46. Friis EM, Pedersen KR, Crane PR (1999) Early angiosperm diversification: The diversity of pollen associated with angiosperm reproductive structures in Early Cretaceous floras from Portugal. Ann Missouri Bot Gard 86: 259-296.

47. Friis EM, Crane PR, Pedersen KR (1997) In: Iwatsuki K RP, editor. Evolution and Diversification of Land Plants. New York: Springer Verlag. pp. 121-156.

48. Rydin C, Pedersen KR, Friis EM (2004) On the evolutionary history of Ephedra; Cretaceous fossils and extant molecules. Proc Natl Acad Sci U S A 101: 16571-16576.

49. Friis EM, Raunsgaard Pedersen K, Crane PR (2006) Cretaceous angiosperm flowers: Innovation and evolution in plant reproduction. Palaeogeogr Palaeoclimatol Palaeoecol 232: 251-293.

50. Anderson CL, Bremer K, Friis EM (2005) Dating phylogenetically basal eudicots using rbcL sequences and multiple fossil reference points. Am J Bot 92: 1737-1748.

51. Doyle JA (1992) Revised palynological correlations of the lower Potomac group (USA) and the Cocobeach sequence of Gabon (Barremian-Aptian). Cretaceous Res 13: 337-349.

52. Crepet WL, Nixon KC, Gandolfo MA (2004) Fossil evidence and phylogeny: the age of major angiosperm clades based on mesofossil and macrofossil evidence from Cretaceous deposits. Am J Bot 91: 1666-1682.

53. Magallón S, Crane PR, Herendeen PS (1999) Phylogenetic pattern, diversity, and diversification of eudicots.Annals of the Missouri Botanical Garden: Springer. 297-372 p.

54. Crepet WL, Nixon KC (1998) Fossil Clusiaceae from the Late Cretaceous (Turonian) of New Jersey and implications regarding the history of bee pollination. Am J Bot 85: 1122-1133.

55. Friis EM, Pedersen KR, Crane PR (1992) Esgueira gen. nov., fossil flowers with combretaceous features from the Late Cretaceous of Portugal. Biologiske Skrifter, Det Kongelige Danske Videnskabernes Selskab 41: 1-45.

56. Soejima A, Wen J (2006) Phylogenetic analysis of the grape family (Vitaceae) based on three chloroplast markers. Am J Bot 93: 278-287.

57. Group AP (1998) An ordinal classification for the families of flowering plants. Ann Missouri Bot Gard 85: 531-553.

58. Gilg E, Brandt M (1911) Vitaceae Africanae. Botanischer Jahresbericht 46: 415-557.

59. Gilg E (1896) Vitaceae. In: Engler A, Prantl K, editors. Die Naturlichen Pflanzenfamilien, III. Germany: W. Engelmann Leipzig. pp. 427-456.

60. Planchon JE (1887) Monographie des Ampélidées vrais. In: de Candolle AFPP, de Candolle C, editors. Monographiae Phanaerogamarum. Paris. pp. 305-654.

61. Chen I, Manchester SR (2007) Seed morphology of modern and fossil Ampelocissus (Vitaceae) and implications for phytogeography. Am J Bot 94: 1534-1553.

62. Patil VP, Kumbhojkar MS, Jadav AS (1980) Chromosome numbers in the family Vitaceae. Curr Sci 49: 37-38.

63. Mullins MG, Bouquet A, Williams LE (1992) Biology of the grapevine; Mullins MG, editor. Cambridge: Cambridge University Press. 239 p.

64. Kubitzki K (2007) The Families and Genera of Vascular Plants; Kubitzki K, editor: Springer.

65. Li H (1992) On the origin of Stachyuraceae. Acta Bot Yunnan 5: 59-64.

66. Jackson RS (2008) Wine Science: Principles and Applications (Food Science and Technology). 751 p.

67. Darlington CD, Wylie AP (1995) Chromosome Atlas of Flowering Plants; Ltd GAU, editor. London. 397 p.

68. Ma JS, Brach AR, Liu QR (1999) A revision of the genus Tripterygium (Celastraceae). Edinb J Bot 56: 33-46.

69. Gu XF (2005) In vitro induction of tetraploid plants from diploid Zizyphus jujuba Mill. cv. Zhanhua. Plant Cell Rep 24: 671-676.
